# On Flare-CME Characteristics from Sun to Earth Combining Remote-Sensing Image Data with *In Situ* Measurements Supported by Modeling

**DOI:** 10.1007/s11207-017-1112-5

**Published:** 2017-07-03

**Authors:** Manuela Temmer, Julia K. Thalmann, Karin Dissauer, Astrid M. Veronig, Johannes Tschernitz, Jürgen Hinterreiter, Luciano Rodriguez

**Affiliations:** 1 Institute of Physics, University of Graz, Graz, Austria; 2 Solar–Terrestrial Center of Excellence, SIDC, Royal Observatory of Belgium, Brussels, Belgium

**Keywords:** CMEs, Flares, Dynamics, Magnetic fields, Corona, Interplanetary space, *In situ* data

## Abstract

We analyze the well-observed flare and coronal mass ejection (CME) from 1 October 2011 (SOL2011-10-01T09:18) covering the complete chain of effects – from Sun to Earth – to better understand the dynamic evolution of the CME and its embedded magnetic field. We study in detail the solar surface and atmosphere associated with the flare and CME using the *Solar Dynamics Observatory* (SDO) and ground-based instruments. We also track the CME signature off-limb with combined extreme ultraviolet (EUV) and white-light data from the *Solar Terrestrial Relations Observatory* (STEREO). By applying the graduated cylindrical shell (GCS) reconstruction method and total mass to stereoscopic STEREO-SOHO (*Solar and Heliospheric Observatory*) coronagraph data, we track the temporal and spatial evolution of the CME in the interplanetary space and derive its geometry and 3D mass. We combine the GCS and Lundquist model results to derive the axial flux and helicity of the magnetic cloud (MC) from *in situ* measurements from *Wind*. This is compared to nonlinear force-free (NLFF) model results, as well as to the reconnected magnetic flux derived from the flare ribbons (flare reconnection flux) and the magnetic flux encompassed by the associated dimming (dimming flux). We find that magnetic reconnection processes were already ongoing before the start of the impulsive flare phase, adding magnetic flux to the flux rope before its final eruption. The dimming flux increases by more than 25% after the end of the flare, indicating that magnetic flux is still added to the flux rope after eruption. Hence, the derived flare reconnection flux is most probably a lower limit for estimating the magnetic flux within the flux rope. We find that the magnetic helicity and axial magnetic flux are lower in the interplanetary space by ∼ 50% and 75%, respectively, possibly indicating an erosion process. A CME mass increase of 10% is observed over a range of ${\sim}\,4\,\mbox{--}\,20~\mathrm{R}_{\odot }$. The temporal evolution of the CME-associated core-dimming regions supports the scenario that fast outflows might supply additional mass to the rear part of the CME.

## Introduction

Since the launch of the *Solar Terrestrial Relations Observatory* (STEREO: Howard *et al.*, 2008), the Sun-Earth distance range is covered as never before. With three eyes viewing the Sun from different vantage points, new insights about the initiation and subsequent propagation of coronal mass ejections (CMEs) in the interplanetary space could be gained. Novel methods of 3D reconstructions of CMEs (Thernisien, Vourlidas, and Howard, 2009; Mierla *et al.*, 2010) and with this, more detailed studies of CME-associated solar surface phenomena (*e.g.*, flares or large-scale waves) were pursued, which were able to largely improve the understanding of CMEs (*e.g.* Kienreich, Temmer, and Veronig, 2009; Temmer *et al.*, 2010; Patsourakos and Vourlidas, 2012; Bein *et al.*, 2012). *In situ* measurements at 1 AU show signatures that can be related to CME-associated solar surface signatures, as well as direct observations of CMEs in white light. In this respect, simultaneous on-disk and off-limb observations provide an invaluable source to link remote sensing and *in situ* signatures. Because of their impact and potential geoeffectiveness, Earth-directed CMEs are of special interest. Using the unprecedented multi-viewpoint data sets currently available, we can enhance our knowledge of CME characteristics and their behavior in interplanetary space. Results obtained using on-disk imagery provide valuable information for periods that are limited to single-viewpoint observations.

The close relation between early CME evolution and its relation to solar flares is well acknowledged (*e.g.* Zhang and Dere, 2006; Temmer *et al.*, 2008). Often associated to flares accompanied by CMEs (hereafter flare-CME events) are dark dimming regions observed as decreased emission in the extreme ultraviolet (EUV) and soft X-rays (SXR). These are most probably caused by the expansion and evacuation of plasma as a result of a CME, and are therefore interpreted as low-coronal footprints of CMEs (Hudson and Cliver, 2001). The analysis of dimming regions is of special interest, as the plasma that is depleted from the corona may reflect the mass that is fed into the CME, maybe over hours (*e.g.* see also Zarro *et al.*, 1999; Harra and Sterling, 2001). Hence, characteristic CME properties may be derived from the dimming evolution (*e.g.* Cheng and Qiu, 2016). Qiu *et al.* (2007) derived the total magnetic reconnection flux in the low corona for flare-associated CMEs and their dimming regions, and compared it to the corresponding magnetic flux in magnetic clouds (MC) at 1 AU. For a sample of nine events, these authors found that the reconnection flux in the flare is related to the magnetic flux of the MC. However, a straightforward comparison between flare characteristics or dimming regions on the solar surface with off-limb measurements or *in situ* counterparts is not an easy task, as unknown processes causing the dimming (Mandrini *et al.*, 2007) or projection effects from single-spacecraft views (Dissauer *et al.*, 2016) may lead to erroneous interpretations.

Early studies linking filament and MC characteristics were successfully performed by Bothmer and Schwenn (1998), who related interplanetary magnetic properties of MCs to filament orientation and handedness at the Sun. The helicity of an erupting flux rope is assumed to be conserved during the CME propagation in interplanetary space, enabling us to link MCs observed *in situ* to their solar sources (*e.g.* Mandrini *et al.*, 2005; Dasso *et al.*, 2005; Rodriguez *et al.*, 2008). With the power of multi-spacecraft data, revealing remote sensing as well as *in situ* data from different vantage points, we are able to have an even more detailed look on the different aspects of CMEs, their interplanetary propagation behavior, and associated *in situ* signatures (*e.g.* Rodriguez *et al.*, 2011; Kilpua *et al.*, 2013; Möstl *et al.*, 2014). More comprehensively, a variety of case studies linked the different aspects of flare-CME events from Sun to Earth in more detail. For instance, Möstl *et al.* (2008) focused on the comparison between the magnetic flux derived from flare reconnection and *in situ* data. Bisi *et al.* (2010) performed an extensive study using multi-instrument data for the analysis of a CME-associated source region that was simulated from vector magnetic field data driven by artificial horizontal flux emergence. In a recent study, Patsourakos *et al.* (2016) tracked the cause of a strong space weather event, in particular focusing on the near-Sun magnetic field strength from which the geoeffectiveness might be assessed.

In the present article we investigate the centrally located on-disk flare-CME event of 1 October 2011 starting at 09:18 UT (SOL2011-10-01T09:18). Compared to already existing studies, we bring new aspects into the dynamic evolution of a CME and its embedded magnetic field by analyzing the solar source region in detail using nonlinear force-free and finite-volume helicity modeling, and deriving the reconnected flux from the CME-associated flare ribbons and dimming areas. In a novel approach we attempt to combine model results from 3D reconstructions of the CME close to the Sun with *in situ* models for obtaining the magnetic field characteristics of the associated MC. We compare the results derived from remote-sensing imagery and *in situ* measurements and discuss the relationship between the parameters.

## Data and Methods

We investigate the flare-CME event of 1 October 2011 in detail. The CME event is launched from NOAA active region (AR) 11305 located at N10W08, associated with an M1.2 GOES class flare (start: 09:18 UT, minor peak: 09:37 UT, major peak: 10:00 UT, end: 10:17 UT).

### Flare Energetics

For the flare evolution, we study full-disk $\text{H}\upalpha $ filtergrams from the *Kanzelhöhe Observatory for Solar and Environmental Research* (KSO) with a temporal cadence of about 6 s that cover the time range 09:18 UT until 11:00 UT (Pötzi *et al.*, 2015). Together with the information of the magnetic field from the 720 s line-of-sight (LOS) magnetograms of the *Helioseismic and Magnetic Imager* onboard the *Solar Dynamics Observatory* (SDO/HMI: Scherrer *et al.*, 2012; Schou *et al.*, 2012; Hoeksema *et al.*, 2014), we derive magnetic reconnection rates from the separation of flare ribbons observed in $\text{H}\upalpha $. The $\text{H}\upalpha $ images are normalized and coaligned to the first image (with north up and derotated to the reference time 09:18 UT). The magnetic field maps are binned to the pixel scale of the $\text{H}\upalpha $ filtergrams using Interactive Data Language (IDL) software (coreg_map.pro). For the alignment between $\mbox{H} \upalpha $ images and the magnetograms, HMI continuum images are used.

**Figure 1:**
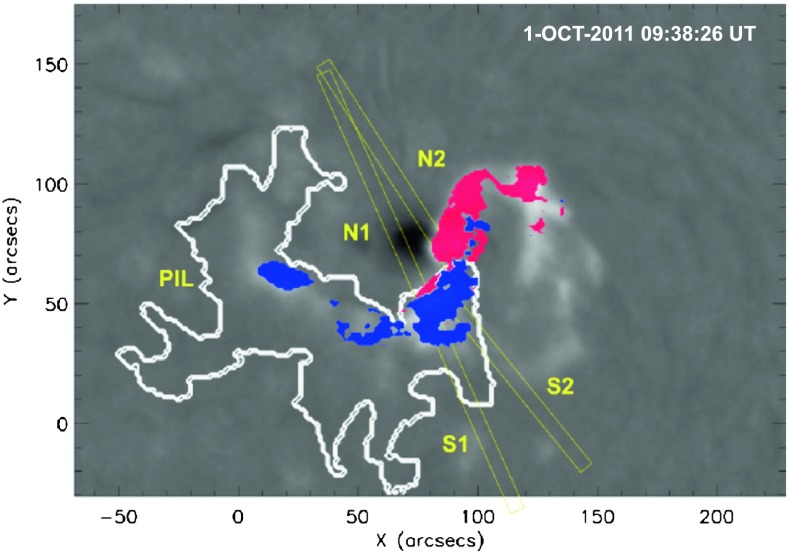
KSO $\mathrm{H}\upalpha$ filtergram showing the flare before it reaches its maximum intensity. The polarity inversion line (PIL) is shown as a white line, different directions (tracking paths: N1, N2 for the northern part and S1, S2 for the southern part) along which the ribbon main motion is tracked are shown with yellow rectangles. The bright flare pixels cumulated until the time of the image shown (09:38:26 UT) are shown as blue (positive polarity) and red areas (negative polarity).

As shown in Figure 1, we derive the flare ribbon separation speed from intensity profiles calculated along rectangular slices oriented perpendicularly to the photospheric inversion line (PIL) along two directions within each magnetic polarity (tracking paths: N1, N2, S1, and S2; see Figure 1). At each time step, the intensity profile of each slice is fitted with a Gaussian function leading to a distance-time diagram. The time derivative of the polynomial fit of the derived distance-time curve is calculated to obtain the ribbon velocity and, hence, the local reconnection rate (Temmer *et al.*, 2007).

The evolution of the flare ribbons provides us with important information on the coronal magnetic reconnection process in solar flare-CME events. Assuming translational symmetry in the flaring arcade that is built up behind the erupting CME, the reconnected electric field in the corona, $E_{\mathrm{c}}$, can be derived from the local ribbon flare separation speed, $v_{\mathrm{r}}$, away from the polarity inversion line together with the underlying normal component, $B_{\mathrm{n}}$, of the photospheric magnetic field at the flare-ribbon location, as $E_{\mathrm{c}} = v_{\mathrm{r}} B_{\mathrm{n}}$ (*cf.* Priest and Forbes, 1986). If the flare does not occur too far from the disk center, the normal component $B_{\mathrm{n}}$ can be well approximated by the LOS field as measured by SDO/HMI. Forbes and Lin (2000) generalized this relation to three dimensions, showing that the rate at which magnetic flux is swept by the flare ribbons relates to a global reconnection rate. Assuming that the change in the photospheric field during the flare is small, this global reconnection rate can be determined from the observations as

1 $$ \dot{\varphi }(t) = \frac{\mathrm{d}\varphi }{\mathrm{d}t} \approx \frac{\partial }{ \partial t}\int B_{\mathrm{n}}(a) \,\mathrm{d}a , $$

with $\varphi $ the magnetic flux, $\mathrm{d}a$ the newly brightened flare area at each instant, and $B_{\mathrm{n}}$ the normal component of the photospheric magnetic field strength underlying $\mathrm{d}a$. This relation basically reflects the conservation of magnetic flux from the coronal reconnection site to the lower atmosphere, where the flare ribbons are observed (Forbes and Lin, 2000).

### Coronal Dimming

We distinguish two different types of dimming regions, core or twin dimmings, and secondary or remote dimmings (see, *e.g.*, Mandrini *et al.*, 2007). *Core* dimmings are found in the form of stationary (long-lived) regions of strongly reduced EUV emission and are closely located to the CME eruption site. Being located in regions of opposite magnetic polarity, they presumably resemble the cross-sectional area of the erupting flux rope footpoints at low coronal heights. Remote dimmings are observed over larger areas, extending to significant distances away from the eruption site, in the form of reduced EUV emission (although not as pronounced as in core-dimming regions).

We calculate the coronal dimming evolution from SDO/*Atmospheric Image Assembly* (AIA: Pesnell, Thompson, and Chamberlin, 2012; Lemen *et al.*, 2012) data in several wavelengths (most sensitive to quiet coronal temperatures around ${\approx}\,0.6\,\mbox{--}\,2 \times 10^{6}~\mbox{K}$, *i.e.* 171 Å, 193 Å, and 211 Å). The time series covers 12 h from the reference time 09:14 UT. We use high-cadence (12 s) observations from 09:14 until 11:14 UT and a successively reduced cadence (1, 5, and 10 min) for the rest of the time series. The dimming regions are identified by applying a thresholding technique on logarithmically scaled base ratio images. A pixel is flagged as a dimming pixel if its logarithmic relative intensity is lower than −0.5 compared to its pre-event value. As an indication of the core-dimming regions, we use the 10% pixels in the dimming region that revealed the largest absolute change in intensity below a certain threshold intensity. Naturally, applying the thresholding technique to the coronal images covering different temperature regimes (wavelength bands) results in different tracked extents of the dimming regions. Visual inspection suggests that the dimming areas are tracked best in 211 Å, which is also supported by previous studies (*e.g.* Robbrecht and Wang, 2010; Kraaikamp and Verbeeck, 2015).

For the magnetic field information we use the 720 s LOS magnetogram of SDO/HMI at the beginning of the event. All data were prepared using standard SolarSoft IDL software (aia_prep.pro, hmi_prep.pro), filtered for constant exposure time, and differentially derotated to the reference time. Based on this, we study the time evolution of the area of the coronal dimming regions and calculate the magnetic flux involved in the total dimming regions, only considering pixels with a magnetic field strength $|B_{i}|>10~\mbox{G}$, *i.e.* above the HMI noise level. We note that values given for the dimming flux are derived from 211 Å image data using the arithmetic mean over positive and negative polarity.

### Coronal Magnetic Field Modeling

The 3D coronal magnetic field configuration in and around NOAA 11305 was modeled based on full-disk vector magnetic field observations from SDO/HMI. The hmi.B_720s data series provides the total field, inclination, and azimuth on the entire solar disk. The azimuth is provided with the 180^∘^ ambiguity already resolved in strong-field regions (using a minimum-energy method). For weak-field regions, we apply a so-called random disambiguation method, using the software tools provided by JSOC.^1^ From the field, inclination, and disambiguated azimuth, we retrieve the image-plane components of the magnetic field vector, *i.e.* the LOS and transverse field. In order to account for projection effects, we deproject the image-plane data to a heliographic coordinate system, *i.e.* we derive the true vertical and horizontal field components, following Gary and Hagyard (1990). A subfield of these optimized full-disk magnetic field data, covering the flaring AR as well as its nearest quiet-Sun surrounding, is used as an input to the nonlinear force-free (NLFF) coronal magnetic field modeling method (for details see Wiegelmann and Inhester, 2010, and Section 2.2.1 of DeRosa *et al.*, 2015). We list two important controlling parameters proposed in the literature (*e.g.* Wheatland, Sturrock, and Roumeliotis, 2000; Schrijver *et al.*, 2006) in order to quantify the goodness of the obtained NLFF coronal magnetic field solution. For the current-weighted average of the sine (CW sin) of the angle between the modeled magnetic field and the electric current density, we find $\text{CW}\sin\approx 0.1$. For the volume-averaged fractional flux we find $\langle |f_{i}|\rangle \approx 10^{-4}$. For a perfectly force-free and solenoidal solution, one would obtain $\mathrm{CW} \sin=0$ and $\langle |f_{i}|\rangle =0$. Using the 3D NLFF field as an input, we employ the finite-volume helicity method of Thalmann, Inhester, and Wiegelmann (2011) in order to estimate the relative helicity of the CME source region.

### CME Morphology and Kinematics

To derive the entire kinematical profile of the CME evolution, we study combined EUV and white-light data from different vantage points using the *Sun Earth Connection Coronal and Heliospheric Investigation* (SECCHI: Howard *et al.*, 2008) instrument suite onboard STEREO, as well as the *Large Angle and Spectroscopic Coronagraph* data (Brueckner *et al.*, 1995) onboard SOHO. On 1 October 2011, the separation angle for STEREO A–Earth and STEREO B–Earth was $104.3^{\circ }$ and $97.5^{\circ }$, respectively, perfectly suited to derive reliable CME kinematics for an event centrally located on the solar disk from Earth view.

To estimate the CME geometry, its main propagation direction, and deprojected bulk speed, we use the graduated cylindrical shell (GCS) reconstruction method (Thernisien, Howard, and Vourlidas, 2006; Thernisien, Vourlidas, and Howard, 2009), which assumes an ideal flux rope to forward fit the appearance of the CME in coronagraph white-light data. The CME early evolution is determined by manually tracking its front part along its main propagation direction, using SECCHI/*Extreme Ultraviolet Imager* (EUVI) (with a field-of-view (FOV) up to $1.7~\mbox{R}_{\odot }$), COR1 (FOV of $1.4\,\mbox{--}\,4.0~\mbox{R}_{\odot }$) and COR2 (FOV of $2.5\,\mbox{--}\,15.0~\mbox{R}_{\odot }$) image data from STEREO A and B. In addition, we use the stereoscopic data in order to calculate the 3D mass of the CME using the method described in Colaninno and Vourlidas (2009). Following Bein *et al.* (2013), we derive the 3D CME mass evolution corrected for occulter effects over the distance range $1\,\mbox{--}\,20~\mbox{R}_{\odot }$.

The interplanetary CME propagation from Sun to Earth orbit is tracked along the main propagation direction of the CME, applying the SATPLOT software tool^2^ available in IDL SolarSoft. The SATPLOT tool delivers j-maps for combined COR2, *Heliographic Imager* 1 (HI1) (FOV $4.0\,\mbox{--}\,24.0^{\circ }$) and *Heliographic Imager* 2 (HI2) (FOV $18.7\,\mbox{--}\,88.7^{\circ }$) white-light data making it easy to measure the elongation angle of the CME under study. The measured elongation-time profile is converted into a radial distance profile using the propagation direction and angular width obtained from the GCS reconstruction. To obtain a range of possible propagation directions, we use several different conversion methods, including Fixed-Phi (FP), Harmonic Mean (HM) and Self-Similar Expansion (SSE), as described in Sheeley *et al.* (1999), Lugaz, Vourlidas, and Roussev (2009), Davies *et al.* (2012), respectively. To calculate the CME speed and acceleration profile from the time-distance data, we apply the regularization method as described in Temmer *et al.* (2010).

### *In situ* CME Characteristics

To correctly identify the *in situ* signatures of the CME (the interplanetary CME or ICME) at Earth orbit, we use a drag-based model (DBM) in order to simulate its interplanetary propagation along the main propagation direction (Vršnak and Žic, 2007; Vršnak *et al.*, 2013). As an input, we use the CME initial speed, distance, and angular width as obtained from the GCS reconstruction. From the results we estimate the time range most suitable for studying the related ICME characteristics. We investigate the *in situ* plasma and magnetic field using one-minute resolution *Wind* data (Lin *et al.*, 1995; Lepping *et al.*, 1995). We apply a Lundquist force-free cylindrical fit (hereafter referred to as Lundquist model; see Lundquist, 1950) to the *in situ* magnetic field data in order to reconstruct the properties of the ICME flux rope, including its interplanetary orientation, radius, and axial field strength. These are then used to calculate its axial magnetic flux and helicity following DeVore (2000).

## Results

### Source Region Characteristics: Pre-Flare Structure

**Figure 2:**
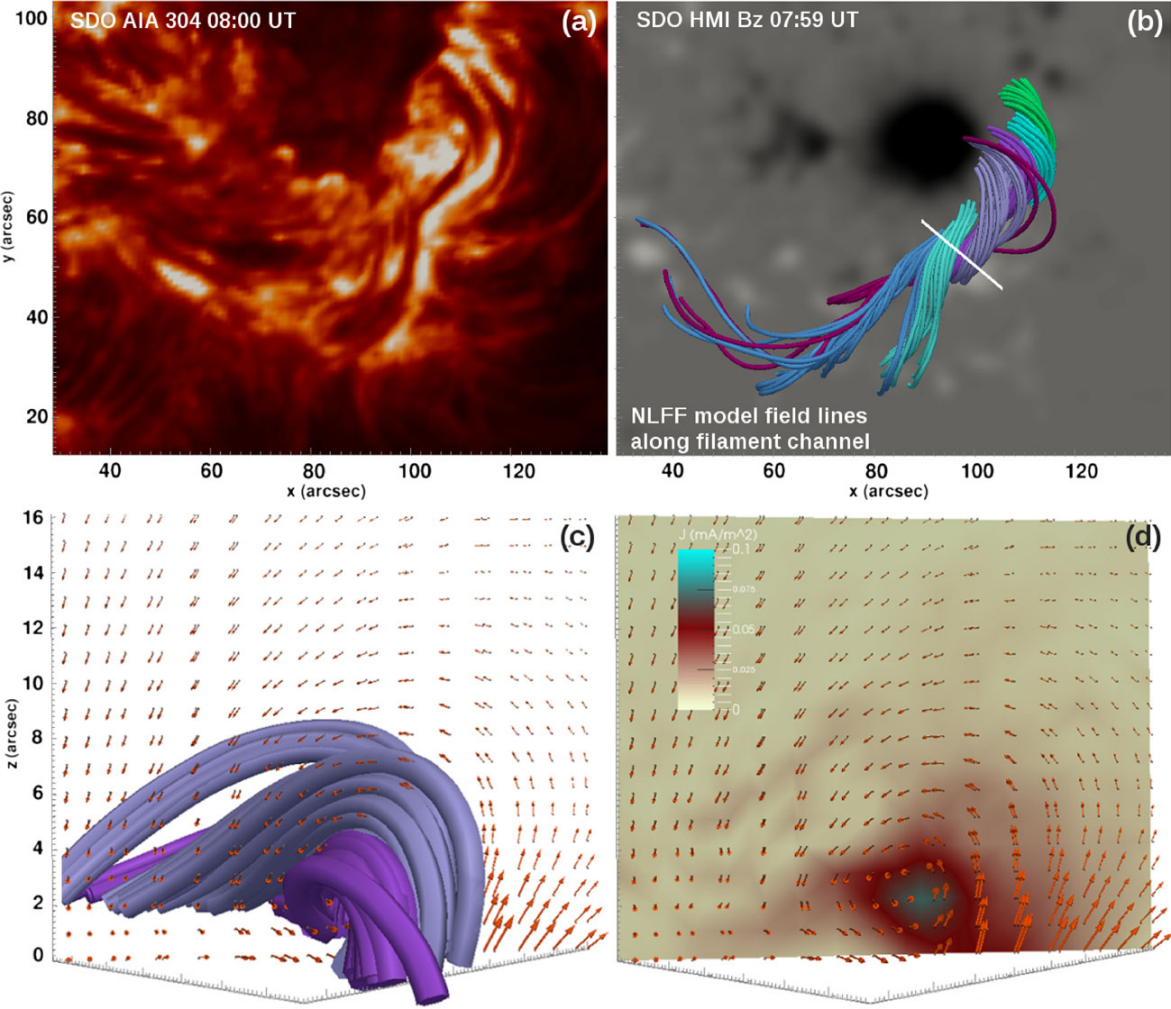
(a) Central filament channel as observed in SDO/AIA 304 Å around the main sunspot of NOAA 11305 before the flare-CME on 1 October 2011. (b) NLFF model magnetic field lines outlining the observed filament channel (colors are for better visibility only). The color-coded background resembles the SDO/HMI vertical magnetic field, scaled to ${\pm}\,2~\mbox{kG}$. (c) Orientation of the coronal magnetic field (orange arrows) in a vertical cut through the model volume above the path outlined as a white solid line in (b). The light and dark violet model field lines are shown as in (b). (d) Orientation of the coronal magnetic field as in (c), but with the magnitude of the total electric current density shown as color-coded background.

The initial conditions for the eruption are derived from the pre-flare NLFF model computed at 07:59 UT. The NLFF coronal magnetic model shows highly twisted magnetic fields along the main PIL (Figure 2b) that clearly outline the dark filament observed in AIA 304 Å (Figure 2a). Projected into a vertical plane roughly perpendicular to the main axis of the filament, the coronal magnetic field vector exhibits a counter-clockwise pattern, *i.e.* a left-handed sense (orange arrows in Figure 2b – c). The NLFF model field lines (violet and purple lines in Figure 2c) warp around a central axis at an approximate height of 3 arcsec (${\approx}\, 2~\mbox{Mm}$) above the photospheric level, characterized by the strongest electrical current density (Figure 2d). These properties are consistent with those of a coronal flux rope (*e.g.* Filippov *et al.*, 2015). The total unsigned axial flux within the flux rope is $\Phi_{\text{ax fr}} = 1.1 \times 10^{21}$ Mx (estimated from the magnetic flux penetrating the vertical plane shown in Figures 2c – d). Using the 3D NLFF field as an input, we estimate the relative helicity of the AR core that hosts the flux rope as $H_{\mathcal{V}}\approx -3.9\times 10^{42}~\mbox{Mx}^{2}$. The relative helicity is a measure of how strongly a field is twisted and/or entangled with respect to a reference potential field (of vanishing electric current and helicity). Its sign arises from the negative contribution of the left-handed fields to the AR helicity budget (see, *e.g.*, the review by Démoulin, 2007).

### Source Region Characteristics: Eruptive Phase

#### Morphology of the Two-Phase Filament Eruption

**Figure 3:**
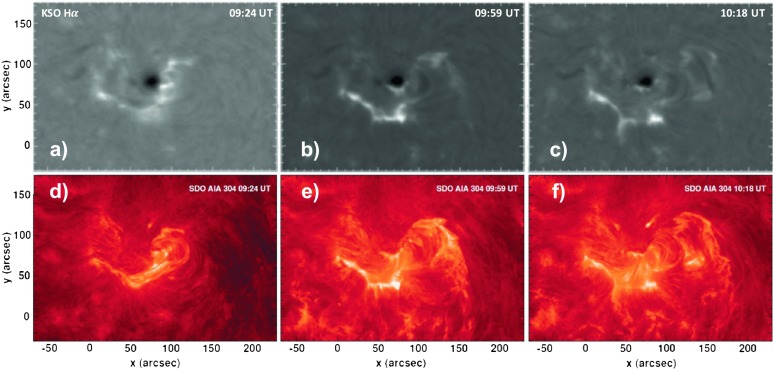
Sequences of KSO $\mbox{H}\upalpha $ (top panels) and AIA 304 Å (bottom panels) images displaying the evolutionary stages of the flare, including the impulsive (left), peak (middle), and decay (right) phase.

Figure 3 shows the morphology of the flare as observed in $\mbox{H} \upalpha $ (Figure 3a – c) and AIA 304 Å (Figure 3d – f). First, the filament to the southwest of the sunspot is activated and starts to rise around 09:22 UT (Figure 3d), simultaneous to an initial rise of the observed SXR emission to C6 X-ray level (compare to Figure 6g). In a second step, the structures in the southeast of the sunspot are destabilized (around 09:37 UT) and end in the final eruption that is observed around ∼ 09:59 UT, cotemporal with the SXR emission rising toward the final M1.2 X-ray level. The flare ribbons observed in $\text{H}\upalpha $ show a consistent evolution. Ribbon formation is observed first to the west of the sunspot (Figure 3a), close to the location where the first post-flare loops become visible in EUV (compare to Figure 3d), and evolves toward the southeast as the flare progresses (Figure 3b).

#### Time Evolution of Flare-Induced Ribbons and CME-Induced Dimming

**Figure 4:**
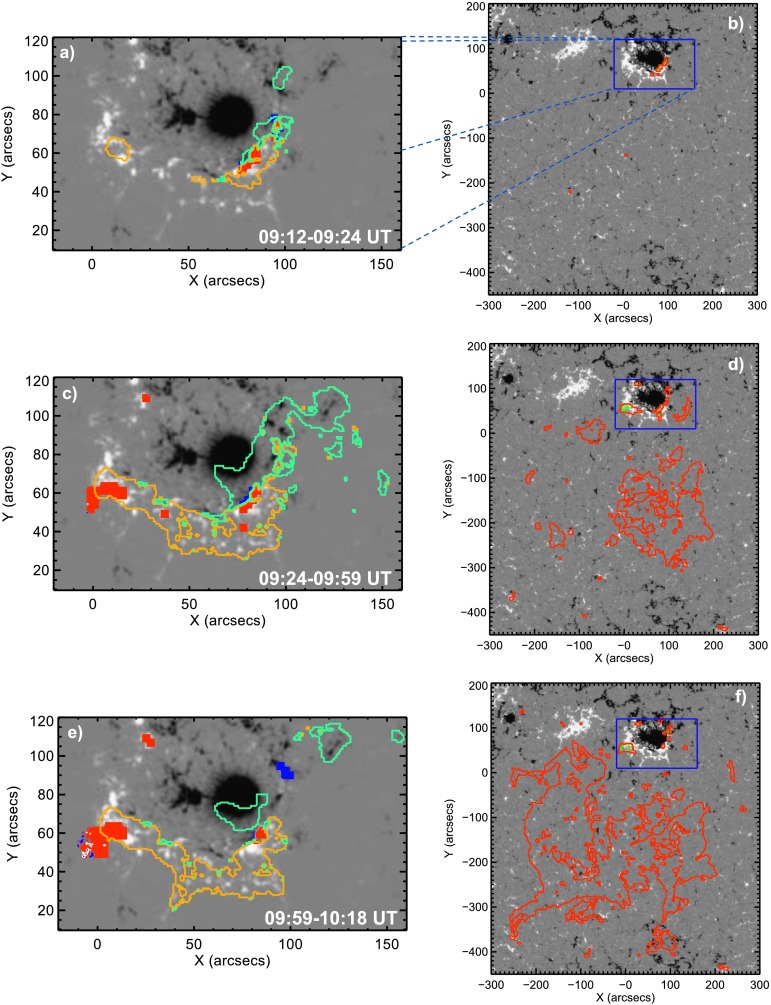
Evolution of flare ribbons and coronal dimming during three different time intervals covering the early impulsive phase (top), late impulsive phase (middle), and the decay phase (bottom) of the flare. Right panels: Area covered by core (green filled contours) and remote (red contours) dimming. The blue rectangle outlines the flare region, shown in the left panels. Left panels: Locations attributed to flare ribbons (cyan and yellow contours for signatures above negative and positive photospheric polarities) and core dimming (blue and red filled contours above negative and positive polarities). The grayscale background resembles the HMI LOS magnetic field at 09:12 UT, scaled to ± 1 kG (left panels) and to ± 0.1 kG (right panels) with black and white representing the negative and positive polarities, respectively.

In Figure 4 we trace both the bright flare-induced ribbon emission and the diminished emission from the CME-induced dimming. From top to bottom, the signatures characteristic for the early impulsive (09:12 – 09:24 UT), late impulsive (09:24 – 09:59 UT), and decay (09:59 – 10:18 UT) phase of the associated flare are shown. The panels of the right column outline the time evolution of the CME-associated coronal dimming, divided into core dimming and remote dimming (see Section 2.2). In the panels of the left column, a close-up of the flare region (marked by the blue rectangle in the right column) is shown. Here, only the core dimming (blue and red filled contours) is shown, together with the locations populated by flare ribbon emission (cyan and yellow contours). During the early impulsive phase, flare ribbons and core-dimming regions (Figure 4a) appear to the southwest of the sunspot, coinciding with the location of the filament observed before the flare (compare to Figure 2a), and marking the footprint of the coronal magnetic field involved in the first phase of the flare. The flare ribbons and core dimming evolve toward the southeast of the sunspot only during the second phase of the flare (the late impulsive phase), coincident with the final eruption of the filament. With the launch of the CME, the formation of pronounced and extended remote dimming areas is initiated (see Figure 4f and compare to Figure 6a – c). As can be seen, this event reveals a complex interplay between flare-brightened areas and core-dimming regions. For this reason we use the core-dimming areas only for qualitative purposes (*cf.* Section 3.3.1).

**Figure 5:**
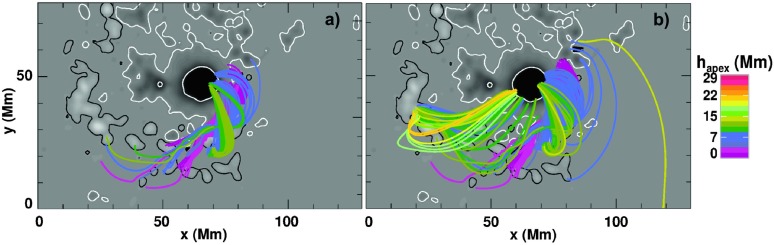
(a) NLFF model field lines calculated from the core-dimming regions, tracked between 09:12 and 09:24 UT (see Figure 4). The field lines are color-coded according to their apex height. The grayscale background resembles the pre-flare vertical magnetic field, scaled to ${\pm}\,2~\mbox{kG}$. (b) NLFF model field lines calculated from the flare pixels tracked in $\mathrm{H}\upalpha $ images for the same time interval as the core dimming shown in (b). Color-coding of the NLFF field lines and background as in (b). Only field lines that close within the field of view and that connect photospheric regions of $B_{z}>10~\mbox{G}$ are shown.

In Figure 5a we show NLFF model field lines traced from the detected core-dimming area (*cf.* Section 2.2) during the early impulsive phase (09:12 – 09:24 UT). This allows us to infer some geometrical properties of the magnetic structure that later developed into the observed CME. It involves twisted fields (a flux rope; compare to Figure 2) with apex heights of ${\lesssim}\,7~\mbox{Mm}$. A comparison with Figure 5b shows the model field lines traced from the flare pixels tracked within the same time interval. In addition to the low-lying magnetic flux rope to the southwest of the sunspot, higher-reaching fields to its southeast (with apex heights up to $\approx 25~\mathrm{Mm}$) were also subject to magnetic reconnection, demonstrating the magnetic connection between the different portions of the AR that were involved in the eruption. Therefore we can assume that the flare-CME process, initiated in the form of a filament eruption to the west of the sunspot (coincident with the early impulsive phase), progressed to the southeast of the sunspot by destabilization of or reconnection with the overlying magnetic configuration in that part of the active region (marking the late impulsive phase of the flare).

#### Relative Timing of Flare- and CME-Associated Features

**Figure 6:**
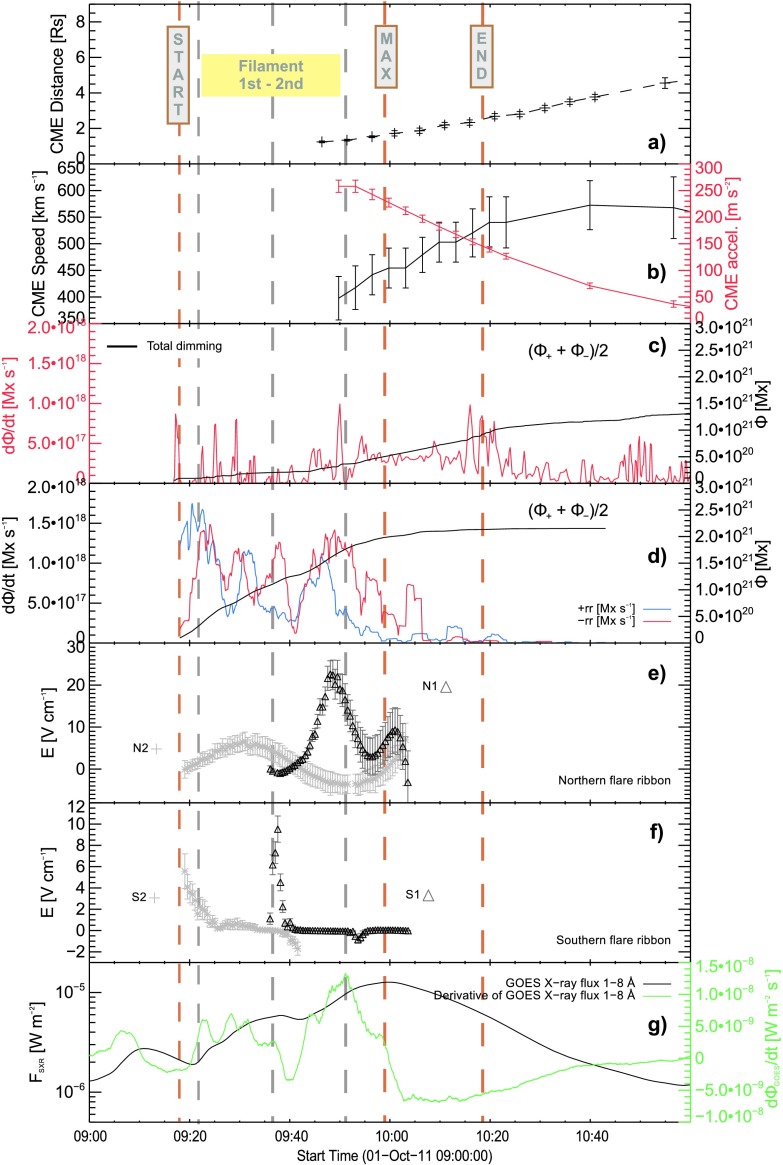
Time evolution of flare- and CME-related parameters, in comparison to the deduced CME kinematics. (a) Distance-, (b) velocity- and acceleration-time profile of the CME. (c) Reconnected magnetic flux deduced from dimming pixels (211 Å) and d) from flare ribbon pixels covering areas of positive (blue) and negative (red) magnetic polarities. Local reconnection rate deduced from the coronal electric field measured along different directions of ribbon motion, normal to the local polarity inversion line, for the (e) northern and (f) southern flare ribbons. (g) GOES soft X-ray flux (black) and its time derivative (green). Yellow vertical dashed lines mark distinct times within the flare process, including the nominal flare start, peak (“MAX”), and end time. Gray vertical dashed lines mark different phases during the filament eruption.

Figure 6 relates different flare- and CME-associated parameters to distinct phases during the flare. The two phases of the filament eruption, including the eruption of the filament to the west of the sunspot and the destabilization of the overlying field to the southeast, match the different phases observed in the GOES X-ray flux and its derivative well (black and green curve in Figure 6g, respectively), the latter being a proxy for the flare-associated hard X-ray emission (Neupert, 1968; Veronig *et al.*, 2002). The time derivative of the magnetic flux associated with the flare ribbon pixels (red and blue curve in Figure 6d, for pixels associated with positive and negative polarity, respectively) reveals major changes throughout the impulsive phase. On the other hand, the time derivative of the magnetic flux associated with the dimming pixels (red curve in Figure 6c) suggests major changes for the time range covering the CME initiation until the CME attains maximum speed. At the end of the decay phase, when the SXR flux again decreased to C X-ray level around 10:45 UT, we find a total accumulated reconnected flux from the flare ribbon evolution of $2.1 \times 10^{21}~\mbox{Mx}$, approximately twice the flux involved in the dimming (${\approx}\, 1.1\times 10^{21}~\mbox{Mx}$ at 10:45 UT).

The local reconnection rate (Figure 6e and f; deduced from the flare ribbon separation velocity and associated magnetic flux; cf. Section 2.1) is distributed in a non-uniform way along the flare ribbon (compare the resulting curves along the two tracking paths N1 and N2 for the northern flare ribbon and along S1 and S2 for the southern flare ribbon, and see also Figure 1), which has also been observed in earlier studies (see *e.g.* Temmer *et al.*, 2007). The velocity and acceleration time profiles of the CME as derived from combined EUV and COR1 measurements (*cf.* Section 2.4) reveal a close relation with the time evolution of (the derivative of) the GOES X-ray flux. The reconnected flux associated with the flaring peaks first, followed by that associated with the dimming, followed by the CME acceleration to its maximum speed (${\sim }\,550~\mbox{km}\,\mbox{s}^{-1}$ at a distance of $4~\mbox{R}_{\odot }$; see Figure 6b).

### CME 3D Characteristics and Kinematical Evolution

#### CME 3D Mass and Near-Sun Kinematics

**Figure 7:**
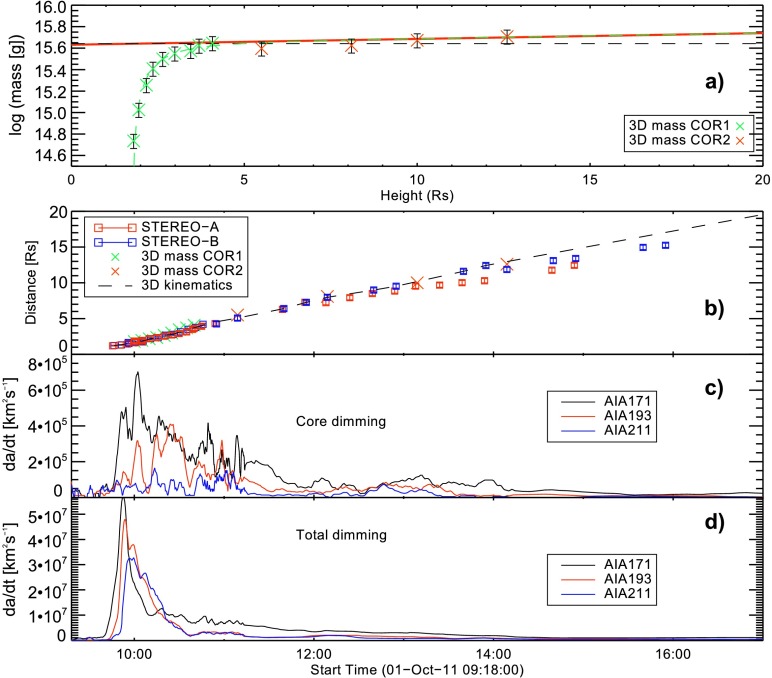
Mass and kinematics of the CME within $20~\mbox{R}_{\odot }$ above the solar surface. (a) 3D mass estimate based on COR1 and COR2 observations. A fit (green dashed line) has been applied to the combined COR1 and COR2 measurements, where only those masses estimated from COR2 have been used that exceed the COR1-based mass estimate at $4~\mbox{R}_{\odot }$. Based on the fit, the 3D mass evolution as function of height corrected for occulter effects (red solid line) and the seed mass (horizontal black dashed line) is calculated. (b) CME distance-time evolution as derived from STEREO A and B white-light images (red and blue squares, respectively) and the deprojected height of the CME front where 3D mass measurements were made (green and red crosses). Cotemporal variation of the (c) core and (d) total dimming area measured from AIA 171, 193, and 211 Å image data.

Figure 7a presents the near-Sun CME 3D mass evolution corrected for occulter effects. The CME kinematics up to a distance of ${\sim}\,20~\mbox{R}_{\odot }$ is given in Figure 7b (*cf.* Figure 6a for the kinematical profile up to ${\sim}\, 5~\mbox{R}_{\odot }$). The results suggest that the flare-associated ejection had a seed mass of $m_{0}=4.4\times 10^{15}~\mbox{g}$, that increased with a rate of $\Delta m =6.1 \times 10^{13}~\mbox{g}\,\mbox{R}_{\odot }^{-1}$. As a result, we estimate the final mass at a distance of $20~\mbox{R}_{\odot }$ as $m_{\mathrm{end}}\sim 5.5\times 10^{15}~\mbox{g}$.

Assuming that the observed increase in mass is due to a continuous mass flow that stems from the coronal regions where the CME footpoints are rooted, we compare the mass evolution to the evolution of the area covered by the CME-related coronal dimming (Figure 7c, d). The time evolution of the total dimming area in three wavelengths (171, 193, and 211 Å; see Figure 7d), shows an effective growth starting at ∼ 09:45 UT that ceased around 10:20 UT. Relatively large variations in the core-dimming area can be traced until ∼ 11:30 UT.

#### CME Geometry

**Figure 8:**
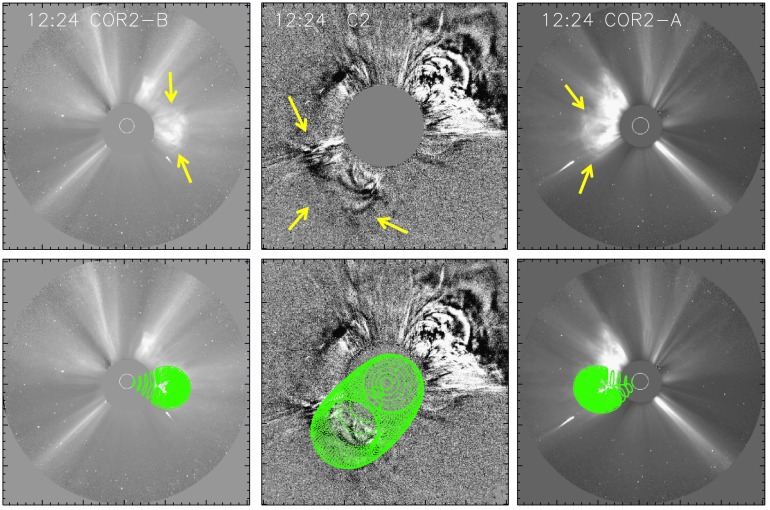
GCS modeling results using the simultaneous view from three spacecraft (STEREO B left, LASCO middle, and STEREO A right) on 1 October 2011. Yellow arrows mark the CME. The CME is directed southeast, with a clear tilt with respect to the ecliptic plane. The FOV of LASCO C2 covers $2\,\mbox{--}\,6~\mbox{R}_{\odot }$, while STEREO A and B cover $2.5\,\mbox{--}\,15~\mbox{R}_{\odot }$, respectively.

The three different vantage points with almost perpendicular separation angles of the two STEREO satellites with respect to SOHO in the Sun – Earth line enable us to reconstruct the 3D geometry of the CME from white-light coronagraphic data. The lower panels of Figure 8 show the best fit (green cones) resulting from the GCS 3D flux rope model when requiring that the boundary of the GCS model flux rope match the outer edge of the CME shape (indicated by yellow arrows in the upper panels) in STEREO B (left), LASCO (middle), and STEREO A (right) white-light images. At $t_{0}=\mbox{13:30~UT}$ we obtain from the GCS model a CME distance of $r_{0}=12~\mbox{R}_{\odot }$, a propagation direction of $\phi_{\mathrm{CME}}=-5^{\circ }$, a speed of $v_{0}=450~\mbox{km}\,\mbox{s}^{-1}$, and a CME half-width of $\lambda =26^{\circ }$. We note that due to the tilt of the reconstructed CME body (${\sim}\,45^{\circ }$), we take an average of the face- and edge-on half-width. All parameters derived from the GCS modeling are summarized in Table 1.

**Table 1:** CME characteristics near the Sun and near Earth using GCS modeling for the CME geometry, 3D mass calculation, and DBM to derive the CME propagation characteristics in interplanetary space and arrival at Earth.

Near Sun		Near Earth (1 AU)	
GCS source region	E08S08	GCS apex radius	$1.7\times 10^{12}~\mbox{cm}$ (0.11 AU)
GCS tilt *vs.* ecliptic	45^∘^	GCS flux rope length (*L*)	$3.9\times 10^{13}~\mbox{cm}$ (2.6 AU)
GCS face-on width	72^∘^	GCS volume	$9.0\times 10^{37}~\mbox{cm}^{3}$
GCS edge-on width	34^∘^	Density^a^	25 – 35 cm^−3^
GCS 3D speed	450 km s^−1^	DBM arrival time	05 Oct. 2011 07:37 UT ± 5 h
3D mass	$5.5 \times 10^{15}~\mbox{g}$	DBM impact speed	$426~\mbox{km}\,\mbox{s}^{-1} \pm 30~\mbox{km}\,\mbox{s}^{-1}$

^a^The density is calculated under the assumption that the mass stays constant beyond $20~\mbox{R}_{\odot }$ and is uniformly distributed within the derived CME volume.

The obtained values are used as input for the DBM in order to model the CME interplanetary propagation. This allows us to compare distinct model parameters with actual *in situ* measurements at Earth orbit. Using a drag value of $\gamma =0.2 \times 10^{-7}$ and an ambient solar wind speed of $w=380~\mbox{km}\,\mbox{s}^{-1}$, we estimate the ICME to arrive at Earth on 5 October 2011 at 07:37 UT (${\pm}\,5~\mbox{h}$), with an impact speed of $426~\mbox{km}\,\mbox{s}^{-1}$ (${\pm}\, 30~\mbox{km}\,\mbox{s}^{-1}$). Comparison with *Wind* observations allows us to determine the arrival of the CME-associated shock at 07:36 UT, with an impact speed of ${\sim}\, 460~\mbox{km}\,\mbox{s}^{-1}$ and followed by a magnetic structure lasting from ∼ 10:00 – 22:00 UT (*cf.* Figure 10). The ICME caused a moderate geomagnetic storm of $\mathit{Dst}=-43~\mbox{nT}$ (Richardson and Cane, 2010, RC list^3^). The modeled and measured results match quite well and reveal that the CME only marginally decelerated on its way from Sun to Earth.

#### Full Kinematical Profile

**Figure 9:**
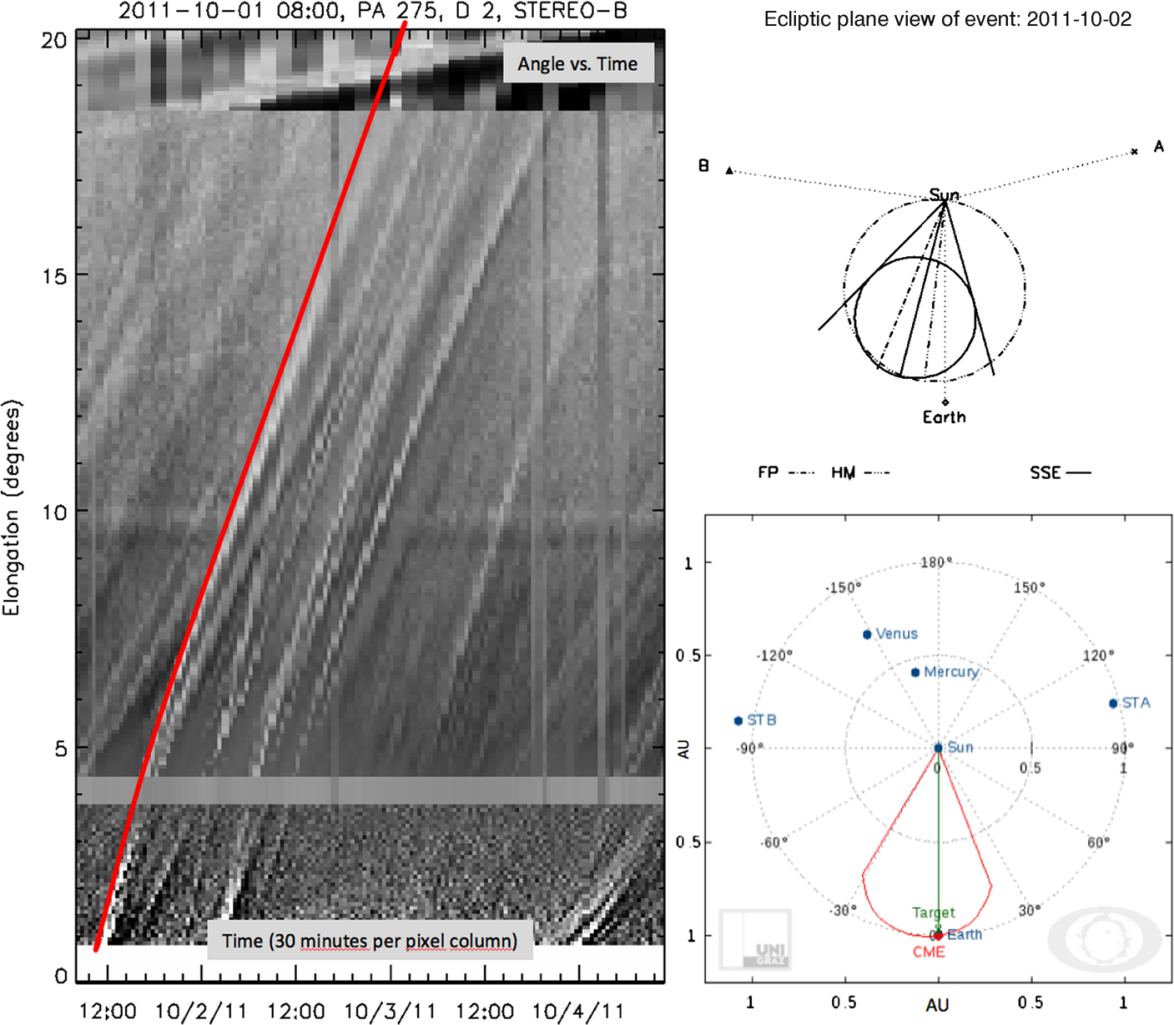
Left: Interplanetary propagation of the CME under study (red line) tracked using SATPLOT j-maps. Top right: Conversion results from the derived elongation angle using several methods with different assumptions on the CME geometry (FP, HM, SSE – for more details see Section 2.4). Bottom right: DBM graphical output (swe.uni-graz.at) using the parameters derived from the GCS model fit as initial values.

**Figure 10:**
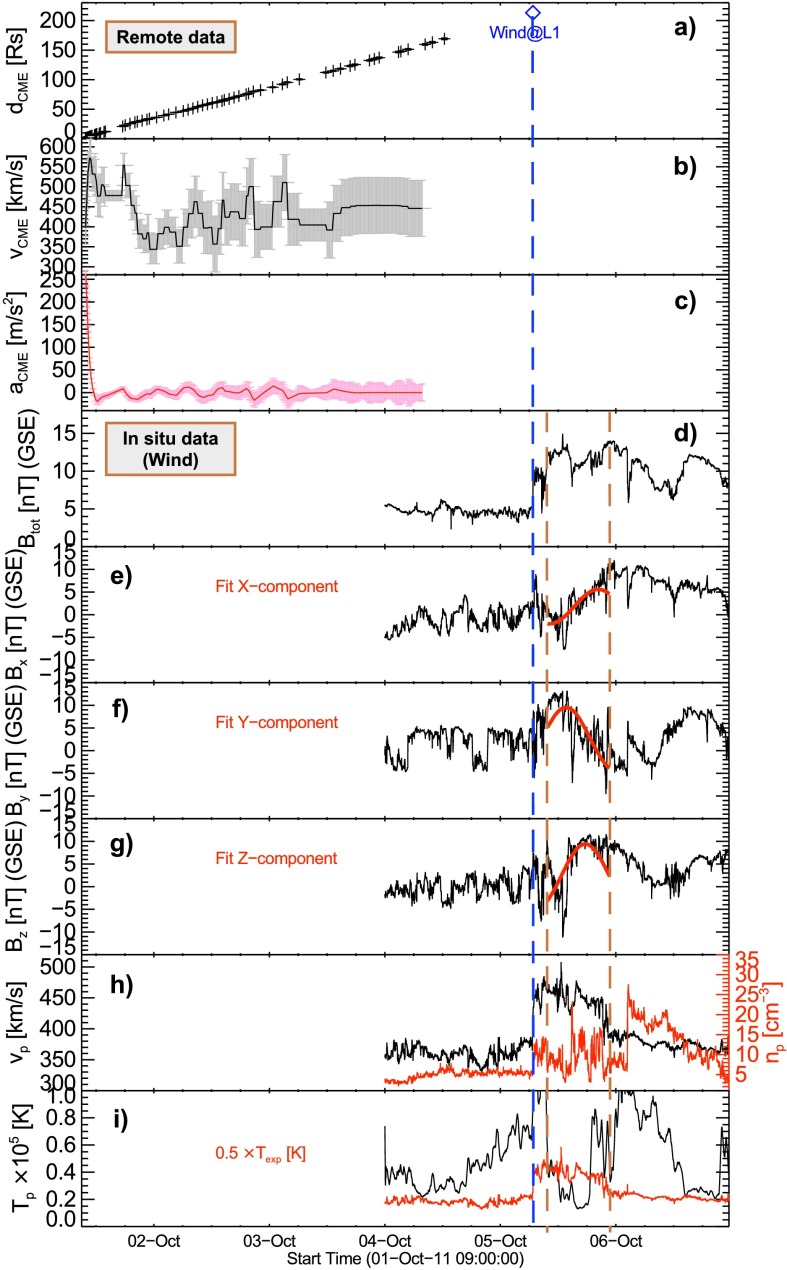
Top three panels: CME kinematics covering the distance range from Sun to the arrival of the CME at the *Wind* spacecraft (blue dashed vertical line), including the (a) measured CME front height from the solar surface (black plus signs), (b) velocity, and (c) acceleration as a function of time. Bottom six panels: *In situ* measurements from *Wind* for the CME showing (d) magnitude and (e – g) direction of the local interplanetary magnetic field in GSE coordinates. The results of the Lundquist fit applied to the *in situ* measurements of the MC covering the time span 5 October 2011 10:00 – 22:00 UT are indicated by red solid lines. (h) Proton speed (solar wind bulk speed, black line), proton density (red line), and (i) proton temperature (black line), together with the expected temperature $T_{\exp}$ for quiet solar wind conditions (red line), based on which the extension of the MC has been determined (brown dashed vertical lines).

We were able to deduce the full kinematical profile of the CME all the way from the low solar corona up to 1 AU based on combined EUV and white-light data. Figure 9 shows the track of the CME in interplanetary space (covered by COR2, HI1, and HI2 data). By applying well-established fitting routines and assuming a constant propagation speed, we deduce the direction of propagation as east $15^{\circ } \pm 10^{\circ }$ ($\mbox{SSE:}-15^{\circ }$, $\mbox{HM:}-7^{\circ }$, $\mbox{FP:}-22^{\circ }$; see top right panel in Figure 9). Importantly, this result is in accordance with the direction of propagation derived from GCS modeling, so that we can safely use the value of $-15^{\circ }$ to convert the measured elongation angle into radial distances and in turn to derive the CME and ICME kinematics, including the speed and acceleration profiles (see Figure 10a – c and Section 2.4 for details). The CME front as observed in HI1 and HI2 cannot be entirely tracked to the distance of L1, but from inspecting Figure 10a, we see that a linear extrapolation of the derived kinematics would match the arrival of the CME at the *Wind* spacecraft well.

The plasma and magnetic field properties measured *in situ* by *Wind*, covering the time range 4 October 2011 00:00 UT to 6 October 2011 24:00 UT, are shown in Figure 10d – i. They suggest that the CME shock-sheath structure arrived at Earth on 5 October at 07:36 UT (indicated by the blue dashed vertical line). Signatures typical of an MC (Burlaga, 1991) were observed, including (i) a rotating magnetic field vector between 10:00 UT and 22:00 UT (see Figure 10e – g), (ii) an enhanced magnetic field strength (Figure 10d), and (iii) a temperature below the typical quiet solar wind temperature (Richardson and Cane, 1995). Applying a Lundquist model to the *in situ* measured data, we deduce an axial field strength of $B_{0}=12.1~\mbox{nT}$, a radius of the MC of $r_{0}=1.75 \times 10^{12}~\mbox{cm}$, and a relative orientation of the MC in interplanetary space (the axis of the embedded flux rope being inclined ${\approx}\,60^{\circ }$ with respect to the Sun–Earth line and ${\approx}\,54^{\circ }$ with respect to the ecliptic plane). Importantly, the inclination with respect to the ecliptic as well as the estimated radius of the MC agree with the corresponding value obtained from GCS modeling (*cf.* Table 1 and Section 3.3.2).

In an original approach, we combine the results obtained from the GCS modeling at 1 AU (*cf.* Table 1) and the cloud parameters derived from Lundquist model of the *in situ* data (*cf.* Table 2) in order to compute the axial flux, $\Phi_{\mathrm{ax}}$, and helicity, $H$, of the MC following DeVore (2000):

2 $$ \Phi_{\mathrm{ax}}=1.4 \cdot B_{0} \cdot r_{0}^{2} , $$

and

3 $$ H=0.6 \cdot H_{\mathrm{s}} \cdot B_{0}^{2} \cdot r_{0}^{3} \cdot L . $$

Here, $H_{\mathrm{s}}$ denotes the helicity sign, which is set to −1, corresponding to the left-handed flux rope deduced from the *in situ* observed ICME signature (see Figure 10d – i), and $L$ is the length of the MC that is calculated from the circumference of the GCS model result viewed face-on at 1 AU (see Table 1). As a result, we obtain for the MC $\Phi_{\mathrm{ax}}=5.2 \times 10^{20}~\mbox{Mx}$ and $H=-1.8\times 10^{42}~\mbox{Mx}^{2}$, in basic agreement within a factor of two with the corresponding values derived for its source region on the Sun (summarized in Table 2, see also Section 3.1).

**Table 2:** *In situ* characteristics using a cylindrical force free model fit (see Figure 10). For comparison we list the parameter values as derived from NLFF model results and the solar source region studies.

*In situ* measurements (*Wind*)		
Shock arrival time	05 Oct. 2011 07:36 UT	RC list
Impact speed	460 km s^−1^	RC list
MC start	05 Oct. 2011 10:00 UT	RC list
MC end	05 Oct. 2011 22:00 UT	RC list
Lundquist model results^a^		
Axial field magnitude	12.1 nT	$B_{0}$
MC radius (GSE)	$1.75\times 10^{12}~\mbox{cm}$ (0.12 AU)	$r_{0}$
*ϕ*	60.7^∘^	angle to Sun–Earth line
Θ	54.1^∘^	angle with ecliptic
*In situ* CME magnetic characteristics		
$\Phi _{\mathrm{ax}}$	$5.2\times 10^{20}~\mbox{Mx}$	
*H*	$-1.8\times 10^{42}~\mbox{Mx}^{2}$	
Solar flare-CME magnetic characteristics		
$\Phi _{\text{ax fr}}$	$1.1\times 10^{21}~\mbox{Mx}$	NLFF
$H_{\mathcal{V}}$	$-3.9\times 10^{42}~\mathrm{Mx}^{2}$	NLFF
Ribbon flux (accumulated)	$2.1\times 10^{21}~\mbox{Mx}$	Hα (09:18 – 10:45 UT)
Dimming flux (accumulated)	$1.1\times 10^{21}~\mbox{Mx}$	193 Å (09:18 – 10:45 UT)
Dimming flux (accumulated)	$1.4\times 10^{21}~\mbox{Mx}$	193 Å (09:18 – 11:30 UT)

^a^We apply the Lundquist model to all *in situ* data between 09:50 and 22:00 UT on 5 October 2011.

## Discussion and Conclusion

We study the CME event from 1 October 2011 in detail. The analysis includes a wealth of data combining remote-sensing and *in situ* instruments to investigate the complete chain of action for the CME eruption and its evolution from Sun to Earth. We obtain detailed information on the solar surface signatures of the associated flare, magnetic field characteristics, and dimming regions that were subsequently related to the *in situ* plasma and magnetic field properties of the CME.

The flare-CME event was associated with a filament eruption that occurred in two steps, starting west of the source AR 11305 and moving toward the southeast. The NLFF results can well explain the process and demonstrate the magnetic connection, showing that in addition to the low-lying magnetic flux rope to the southwest of the sunspot, higher-reaching fields to its southeast were also subject to magnetic reconnection. This is reflected in the CME propagation direction (E15) when compared to the source region coordinates (W08), and also in the location of the remote dimming regions. We conclude that the magnetic flux rope of the CME is fed by two components, low-lying twisted magnetic fields (rooted in core-dimming regions) and sheared overlying magnetic fields (rooted in flare pixels) involved in the eruption. We derive a flare reconnection flux of $2.1\times 10^{21}~\mbox{Mx}$ and a dimming flux of $1.1 \times 10^{21}~\mbox{Mx}$.

From $\text{H}\upalpha $ emission we obtain the magnetic flux injected to the CME flux rope at different stages of eruption. We derive an equal amount of flare reconnection flux during the first impulsive phase of the flare (09:18 – 09:44 UT), *i.e.* before the SXR emission reaches an M X-ray level, and during the flare major impulsive phase (> 09:44 UT). Hence, reconnection processes were well ongoing before the filament started to erupt (09:37 UT), followed by the restructuring of the magnetic field. In comparison, the dimming flux that covered remote areas in the outskirts of the AR shows regions involved in the reconnection process at a later time when the CME had already fully erupted (*cf.* bottom panel of Figure 4). Over the time range 10:45 – 11:30 UT, after the flare ceased, the dimming flux increased from $1.1\times 10^{21}~\mbox{Mx to }1.4 \times 10^{21}~\mbox{Mx}$. This indicates that magnetic flux might have been added to the flux rope through ongoing magnetic restructuring that is too weak to produce visible $\text{H}\upalpha $ flare ribbon emission. Therefore, the value of $2.1\times 10^{21}~\mbox{Mx}$ for the total axial flux is most probably a lower limit. When comparing this to the *in situ* axial magnetic flux of the MC (${\sim}\,0.5\times 10^{21}~\mbox{Mx}$), we find that it is reduced by at least 75% and that the helicity is reduced by a factor of two. This might refer to an erosion of the MC while propagating in interplanetary space (*e.g.* Dasso *et al.*, 2006; Ruffenach *et al.*, 2015). To calculate the helicity from *in situ* data, we took the best estimate of the MC length $L$, as derived from GCS modeling.

The determination of the magnetic flux *in situ* as well as the MC radius and length is prone to substantial errors. This is because the parameters used in its determination are derived from the fitting of an idealized magnetic field model (Lundquist force-free cylindrical fit in our case) to *in situ* data, where the selection of the MC boundaries affects the calculations. Furthermore, *in situ* models rely on a single 1D spacecraft crossing a 3D structure, taking many assumptions into play (Démoulin, Janvier, and Dasso, 2016). The GCS model is also a fit of an idealized CME shape to white-light data. In this respect, we note that the MC radius, an important parameter for calculating the flux and helicity as determined from the *in situ* model fit matches the radius derived from the GCS extrapolated to 1 AU well. For other case studies on this issue that include poloidal flux components, we refer to Mandrini *et al.* (2005), Attrill *et al.* (2006), and Qiu *et al.* (2007).

The temporal profile of core-dimming areas indicates that the observed CME mass increase of 10% in total is supplied by the fast outflow from the core-dimming regions. The CME mass consists of coronal plasma that becomes compressed and moves away from the Sun as a result of the explosive release of magnetic energy. The associated dimming regions map the evacuation of the plasma, with the core dimming marking the CME footpoints and the remote dimming the CME body. In a qualitative approach we attempt to relate the temporal evolution of the core dimming and the CME mass increase. The plasma evacuated from the core-dimming area would be detected in COR2 white light only beyond the occulter radius of $2.5~\text{R}_{\odot }$. Assuming an outflow speed on the order of 100 – 200 km s^−1^ (*e.g.* Zarro *et al.*, 1999; Harra and Sterling, 2001; Tian *et al.*, 2012), the plasma flow could be a detectable part of the CME mass after ∼ 1.5 – 3 h (assuming a detection height beyond $4~\hbox{R}_{\odot }$, see Bein *et al.*, 2013, this would yield 3 – 5.75 h). EUV observations reveal that the major changes in the core dimming end around 11:30 UT. Accordingly, outflows would feed mass into the CME until that time. Taking the propagation time as described above into account, this mass would become visible in the coronagraph white-light data the latest at around ∼ 17:15 UT. The CME apex is at a distance of about $17\,\mbox{--}\,18~\mbox{R}_{\odot }$ at that time. This is consistent with statistical results showing that the increase in CME mass is primarily supplied to the rear part of the CME to distances below $20~\mbox{R}_{\odot }$ (see Bein *et al.*, 2013). This is supported by Bemporad and Mancuso (2010), who observed at a distance of $4.1~\mbox{R}_{\odot }$ continuous outflows in the *Ultraviolet Coronagraph Spectrometer* (UVCS) data over hours after the CME shock propagated through. They concluded that the transit of the CME flank left the coronal magnetic field open over ∼ 6 h, facilitating fast plasma outflow before the corona recovered to the pre-CME configuration that slowed the outflowing plasma down.

We calculated the CME density using the GCS volume derived for the CME apex to be at 1 AU and the observed 3D mass at $20~\mbox{R}_{\odot }$, which is assumed to be conserved. The derived density (25 – 35 cm^−3^) is comparable within a factor of two to the *in situ* measurements (10 – 15 cm^−3^). However, the unknown plasma distribution in a CME volume still leaves many questions open, such as compression during the eruption and subsequent expansion, as well as mass supply from fast outflows like core-dimming regions. We note that the CME mass increase might continue during propagation in interplanetary space through material swept up from the solar wind (“snowplow effect”; Cargill, 2004). In the literature one finds quite high factors on the order of 2 – 3 for the CME mass increase in interplanetary space (*e.g.* Lugaz, Manchester, and Gombosi, 2005; DeForest, Howard, and McComas, 2013). This might have effects on the drag that the CME experiences during its propagation phase. According to the DBM results for the event under study, the drag was of “normal” type, and from the density estimate and comparison to the *in situ* plasma density data, we cannot confirm a substantial mass increase.

Combining model and data at various distance ranges gives us new insight into the CME characteristics as it propagates from Sun to Earth. However, the uncertainties especially in the derived magnetic field parameters and the lack of *in situ* data at close distances to the Sun still leave many questions open. New missions such as *Solar Orbiter* or *Solar Probe Plus* will produce most eligible data sets to further pursue these studies.

## Footnotes

1 The Joint Science Operations Center (JSOC) provides supporting documentation and software for HMI data. For details on the data series and related azimuth disambiguation procedure see http://jsoc.stanford.edu/jsocwiki/FullDiskDisamb.

2
http://hesperia.gsfc.nasa.gov/ssw/stereo/secchi/idl/jpl/satplot/SATPLOT_User_Guide.pdf.

3
http://www.srl.caltech.edu/ACE/ASC/DATA/level3/icmetable2.htm.
